# The tobacco GNTI stem region harbors a strong motif for homomeric protein complex formation

**DOI:** 10.3389/fpls.2023.1320051

**Published:** 2023-11-28

**Authors:** Jennifer Schoberer, Shiva Izadi, Carolina Kierein, Ulrike Vavra, Julia König-Beihammer, Valentina Ruocco, Clemens Grünwald-Gruber, Alexandra Castilho, Richard Strasser

**Affiliations:** ^1^ Department of Applied Genetics and Cell Biology, University of Natural Resources and Life Sciences, Vienna, Austria; ^2^ Core Facility Mass Spectrometry, University of Natural Resources and Life Sciences, Vienna, Austria

**Keywords:** cell biology, glycoengineering, glycosylation, Golgi apparatus, protein-protein interaction, recombinant protein

## Abstract

**Introduction:**

The Golgi apparatus of plants is the central cellular organelle for glycan processing and polysaccharide biosynthesis. These essential processes are catalyzed by a large number of Golgi-resident glycosyltransferases and glycosidases whose organization within the Golgi is still poorly understood.

**Methods:**

Here, we examined the role of the stem region of the *cis*/medial Golgi enzyme N-acetylglucosaminyltransferase I (GNTI) in homomeric complex formation in the Golgi of *Nicotiana benthamiana* using biochemical approaches and confocal microscopy.

**Results:**

Transient expression of the N-terminal cytoplasmic, transmembrane, and stem (CTS) regions of GNTI leads to a block in N-glycan processing on a co-expressed recombinant glycoprotein. Overexpression of the CTS region from Golgi α-mannosidase I, which can form *in planta* complexes with GNTI, results in a similar block in N-glycan processing, while GNTI with altered subcellular localization or N-glycan processing enzymes located further downstream in the Golgi did not affect complex N-glycan processing. The GNTI-CTS-dependent alteration in N-glycan processing is caused by a specific nine-amino acid sequence motif in the stem that is required for efficient GNTI-GNTI interaction.

**Discussion:**

Taken together, we have identified a conserved motif in the stem region of the key N-glycan processing enzyme GNTI. We propose that the identified sequence motif in the GNTI stem region acts as a dominant negative motif that can be used in transient glycoengineering approaches to produce recombinant glycoproteins with predominantly mannosidic N-glycans.

## Introduction

**Figure 1:**
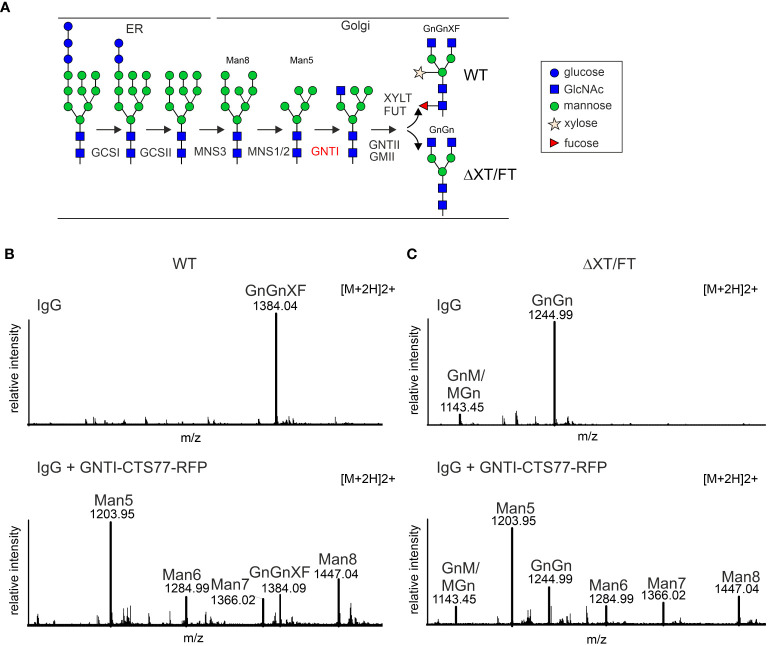
Transient expression of a chimeric protein containing the tobacco GNTI-CTS77 region results in the accumulation of Man_5_GlcNAc_2_ N-glycans on a recombinantly expressed IgG antibody. **(A)** Simplified illustration of the major N-glycan processing steps in plants. GCSI, α-glucosidase I; GCSII, α-glucosidase II; MNS3, ER α-mannosidase; MNS1/2, Golgi α-mannosidase I (two forms, termed MNS1 and MNS2, with redundant function, are present in *Arabidopsis thaliana*); GNTI, β1,2-*N*-acetylglucosaminyltransferase I; GMII, Golgi α-mannosidase II; GNTII, β1,2-*N*-acetylglucosaminyltransferase II; XYLT, β1,2-xylosyltransferase; FUT, core α1,3-fucosyltransferases (two forms, termed FUT11/FUT12, with redundant function, are present in *A. thaliana*). The symbols for the monosaccharides in the illustration are drawn according to the nomenclature from the Consortium for Functional Glycomics (http://www.functionalglycomics.org/). **(B)** MS-spectra of glycopeptides (peptide sequence: EEQYNSTYR, derived by trypsin digestion of the heavy chain polypeptide from human IgG1) showing the GNTI-CTS effect in wild-type (WT) or **(C)** in ΔXT/FT plants. The nomenclature for glycan abbreviations is in accordance with the ProGlycAn system (www.proglycan.com). GnGnXF, GnM/MGn, and GnGn depict complex N-glycans; Man5-Man8 refers to mannosidic N-glycans; Man_5_GlcNAc_2_ to Man_8_GlcNAc_2_.

Glycosylation is an essential co- and post-translational modification in all multicellular organisms. N-glycosylation is initiated in the lumen of the ER by the transfer of a preassembled oligosaccharide to asparagine residues present in the canonical glycosylation sequence Asn-X-Ser/Thr (X any amino acid except proline). The attached N-glycans subsequently undergo trimming and processing from mannosidic-type ones to complex-type ones. The complex N-glycans are generated in the Golgi apparatus, and distinct modifications on these N-glycans contribute to specific functions of the glycoproteins (Strasser, 2022). N-acetylglucosaminyltransferase I (GNTI or MGAT1) is one of the key processing enzymes in the *cis*/medial Golgi, as it initiates the formation of hybrid-type and complex N-glycans by transferring a single GlcNAc residue to Man_5_GlcNAc_2_ (Strasser et al., 1999a). The attached terminal GlcNAc is required for efficient processing by other Golgi-resident N-glycan processing enzymes such as Golgi-α-mannosidase II (GMII, Strasser et al., 2005), N-acetylglucosaminyltransferase II (GNTII, Strasser et al., 1999b), or β1,2-xylosyltransferase (XYLT) (Strasser et al., 2000) (**Figure 1A**). In mammals, GNTI deficiency and subsequent absence of complex N-glycans are embryo-lethal (Ioffe and Stanley, 1994; Metzler et al., 1994). *Oryza sativa* and *Lotus japonicas* GNTI knockout mutants display severe growth defects and problems with reproduction (Fanata et al., 2013; Pedersen et al., 2017). *Arabidopsis thaliana* GNTI-deficient plants are more sensitive to salt stress and show alterations in root hair development (Kang et al., 2008; Frank et al., 2021; Strasser, 2022). Together, these examples highlight the importance of GNTI function for different multicellular organisms, including plants.

Like most Golgi-resident glycosyltransferases, GNTI is a type II transmembrane protein with a characteristic domain organization. It harbors a short N-terminal tail facing the cytosol (C region), a single transmembrane helix (T domain), a stem or spacer region (S region) (together referred to as the CTS region), and a large luminal catalytic domain (Colley, 1997; Schoberer and Strasser, 2011). While different protein parts, including the catalytic domain, may contribute to Golgi localization of glycosyltransferases in mammalian cells (Fenteany and Colley, 2005; Khoder-Agha et al., 2019), the subcellular distribution of tobacco (*Nicotiana tabacum*) GNTI is mediated by its N-terminal CTS region in plants (Essl et al., 1999; Schoberer et al., 2014). The tobacco GNTI-CTS region carries basic amino acids in the cytoplasmic tail that are required for ER export (Schoberer et al., 2009). The single transmembrane domain is involved in sub-Golgi distribution (Schoberer et al., 2019), and the stem region between the transmembrane and catalytic domains mediates GNTI homomeric and heteromeric complex formation (Schoberer et al., 2013; Schoberer et al., 2014). While GNTI homomeric complex formation in plants does not seem to be required for its Golgi localization and catalytic activity (Schoberer et al., 2014), *in vivo* heterodimerization with Golgi α-mannosidase I (MNS1) and possible other *cis*/medial Golgi enzymes is a common feature that is found in mammalian cells (Hassinen et al., 2010; Hassinen and Kellokumpu, 2014) and in plants for glycosyltransferases involved in cell wall polysaccharide biosynthesis (Hoffmann et al., 2021; Zabotina et al., 2021). Here, we examined the role of the GNTI stem region in homomeric complex formation in the Golgi apparatus of plants. We found that a small intrinsically disordered region in a predicted coiled-coil domain within the stem region of tobacco GNTI plays a dominant-negative role when overexpressed in *N. benthamiana*. Expression of the tobacco GNTI-CTS region can be used in transient glycoengineering approaches to generate recombinant glycoproteins with increased amounts of mannosidic N-glycans for functional characterization.

## Results

### Transient overexpression of the tobacco GNTI-CTS77 region alters the N-glycan profile of a co-expressed glycoprotein

In previous studies, we expressed different chimeric glycosyltransferases and examined their effect on Golgi localization and N-glycan processing (Strasser et al., 2009; Castilho et al., 2011; Schoberer et al., 2014). The chimeric proteins carried CTS regions from Golgi-resident plants and mammalian N-glycan processing enzymes fused to catalytic domains of heterologous enzymes such as human β1,4-galactosyltransferase. When we co-expressed chimeric enzymes carrying the tobacco GNTI-CTS region, we observed increased levels of the mannosidic Man_5_GlcNAc_2_ N-glycan on recombinant glycoproteins such as human IgG. To examine this effect in more detail and to avoid possible interference from the enzymatic activity of the attached catalytic domain, we fused the tobacco GNTI-CTS region (amino acids 1 to 77 – GNTI-CTS77) to RFP and transiently co-expressed GNTI-CTS77-RFP with an IgG antibody in *N. benthamiana* leaves. When expressed alone, IgG displayed almost exclusively the commonly found complex N-glycan GlcNAc_2_XylFucMan_3_GlcNAc_2_ (GnGnXF) in wild-type (**Figure 1B**) and GlcNAc_2_Man_3_GlcNAc_2_ (GnGn) in glycoengineered ΔXT/FT plants (**Figure 1C**), which is consistent with results from earlier research (Strasser et al., 2008; Castilho et al., 2018). This glycoengineered line almost completely lacks plant-specific complex N-glycan modifications due to the silencing of *XYLT* and core α1,3-fucosyltransferase (*FUT*) gene expression. When GNTI-CTS77-RFP was co-expressed, the levels of complex N-glycans were drastically reduced in both wild-type and ΔXT/FT plants, while mannosidic N-glycans increased. The main peak was assigned to Man_5_GlcNAc_2_, indicating that overexpression of the GNTI-CTS77 region affects the processing of mannosidic N-glycans into complex ones. A similar N-glycan profile was obtained when the MNS1-CTS (amino acids 1-88) region was co-expressed with IgG (**Supplementary Figure S1**). In contrast, much fewer Man_5_GlcNAc_2_ structures were detected with CTS regions from GMII (GMII-CTS, amino acids 1-92) or XYLT (XYLT-CTS, amino acids 1-90) (**Supplementary Figure S1**), which act later in the N-glycan processing pathway and are likely concentrated in a different Golgi compartment. These findings suggest that the inhibition of complex N-glycan formation is specific for the *cis*/medial Golgi enzyme GNTI and its major interaction partner MNS1 (Schoberer et al., 2014).

### GNTI variants with small deletions in the stem region are located in the Golgi apparatus

**Figure 2:**
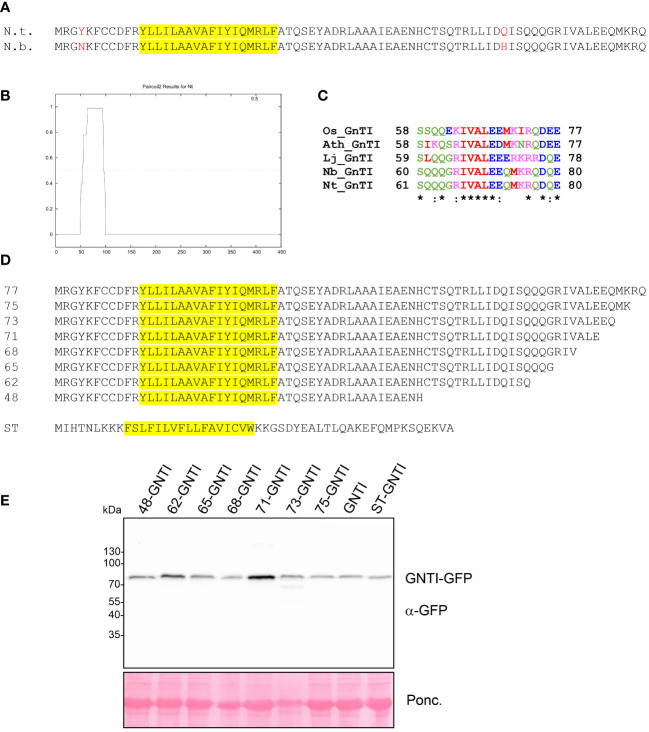
Analysis of GNTI-GFP variants with deletions in the GNTI stem region. **(A)** Sequence comparison of the GNTI-CTS77 regions from *N. tabacum* (N.t.) and *N. benthamiana* (N.b.). The predicted transmembrane domain is highlighted in yellow. **(B)** Coiled-coil domain prediction of the full-length tobacco GNTI amino acid sequence using Paircoil2 (https://cb.csail.mit.edu/cb/paircoil2/paircoil2.html). **(C)** ClustalW alignment of amino acid regions 61-80 (N.t. numbering) from different GNTI sequences (Os, *Oryza sativa*; Ath, *Arabidopsis thaliana*; Lj, *Lotus japonicus*; Nb, *Nicotiana benthamiana*; Nt, *Nicotiana tabacum*). **(D)** Overview of the amino acid sequences of the expressed GNTI-CTS deletion proteins. The amino acid number (length of the CTS) is given for each GNTI-CTS region. ST, CTS region (52 amino acids) from rat α2,6-sialyltransferase. The predicted transmembrane domain is highlighted in yellow. **(E)** Immunoblot analysis of transiently expressed GNTI-GFP variants. Total soluble protein (TSP) extracts from *N. benthamiana* leaves expressing different GNTI-GFP variants were analyzed by immunoblotting at 2 days post-infiltration (dpi) using an anti-GFP antibody. Ponc., Ponceau S-stained membrane.

**Figure 3:**
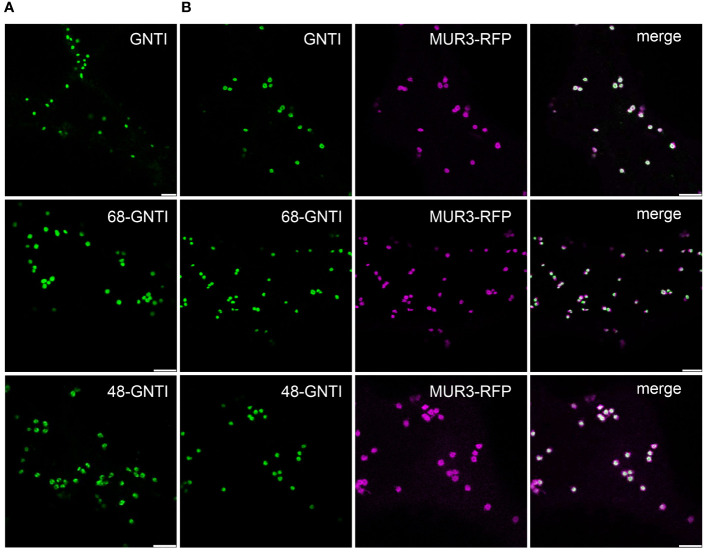
The Golgi localization of GNTI variants with deletions in the stem region is not altered. Confocal microscopy analysis of selected GNTI-GFP variants (GNTI, 68-GNTI, and 48-GNTI) transiently expressed in *N. benthamiana* leaf epidermal cells either **(A)** expressed alone (shown in green) or **(B)** co-expressed with the Golgi marker MUR3-RFP (in magenta). Merged images show the overlay of the signals (in white). Scale bars = 5 µm.

GNTI and MNS1 form homomeric and heteromeric complexes in the Golgi apparatus of plants, with the stem region of tobacco GNTI playing a key role in homomeric complex formation (Schoberer et al., 2013; Schoberer et al., 2014). Thus, we hypothesized that this region contains a sequence motif that governs a dominant-negative effect on endogenous GNTI, resulting in a block in N-glycan processing and a concomitant increase in Man_5_GlcNAc_2_ and other mannosidic structures. The GNTI-CTS regions of tobacco (*N. tabacum*) and *N. benthamiana* are highly conserved and differ only by two amino acid residues (Y4N in the cytoplasmic tail and Q59H in the stem region) (**Figure 2A**). To identify sequence motifs that are potentially involved in protein-protein interaction within the tobacco GNTI stem region (amino acids 30-77), we subjected the full-length tobacco GNTI sequence (446 amino acids) to secondary structure prediction tools. The region from 50 to 90 amino acids was predicted to be a coiled-coil domain, and amino acids 70-73, which are conserved in plant GNTI proteins, were predicted to be an intrinsically disordered region (**Figures 2B, C**, and **Supplementary Figure S2**). Next, we generated constructs for the expression of full-length GNTI with small deletions in the stem region (**Figure 2D**). These GFP-tagged GNTI variants were transiently expressed in *N. benthamiana* and analyzed by immunoblotting. Bands of the expected size were detectable for all chimeric proteins, indicating that the small deletions do not affect GNTI protein expression or stability (**Figure 2E**). Confocal microscopy of selected variants, such as full-length GNTI (77 amino acid-long CTS region), 48-GNTI-GFP (48 amino acid-long CTS region fused to the catalytic domain of GNTI), and 68-GNTI-GFP (68 amino acid-long CTS region fused to the catalytic domain of GNTI), revealed accumulation in Golgi bodies. This was confirmed by co-expression with the Golgi-resident glycosyltransferase MUR3-RFP, which is involved in cell wall polysaccharide synthesis and does not form a complex with GNTI (Schoberer et al., 2019) (**Figure 3**).

### A small, potentially disordered region within the GNTI stem is required for GNTI homomeric complex formation

**Figure 4:**
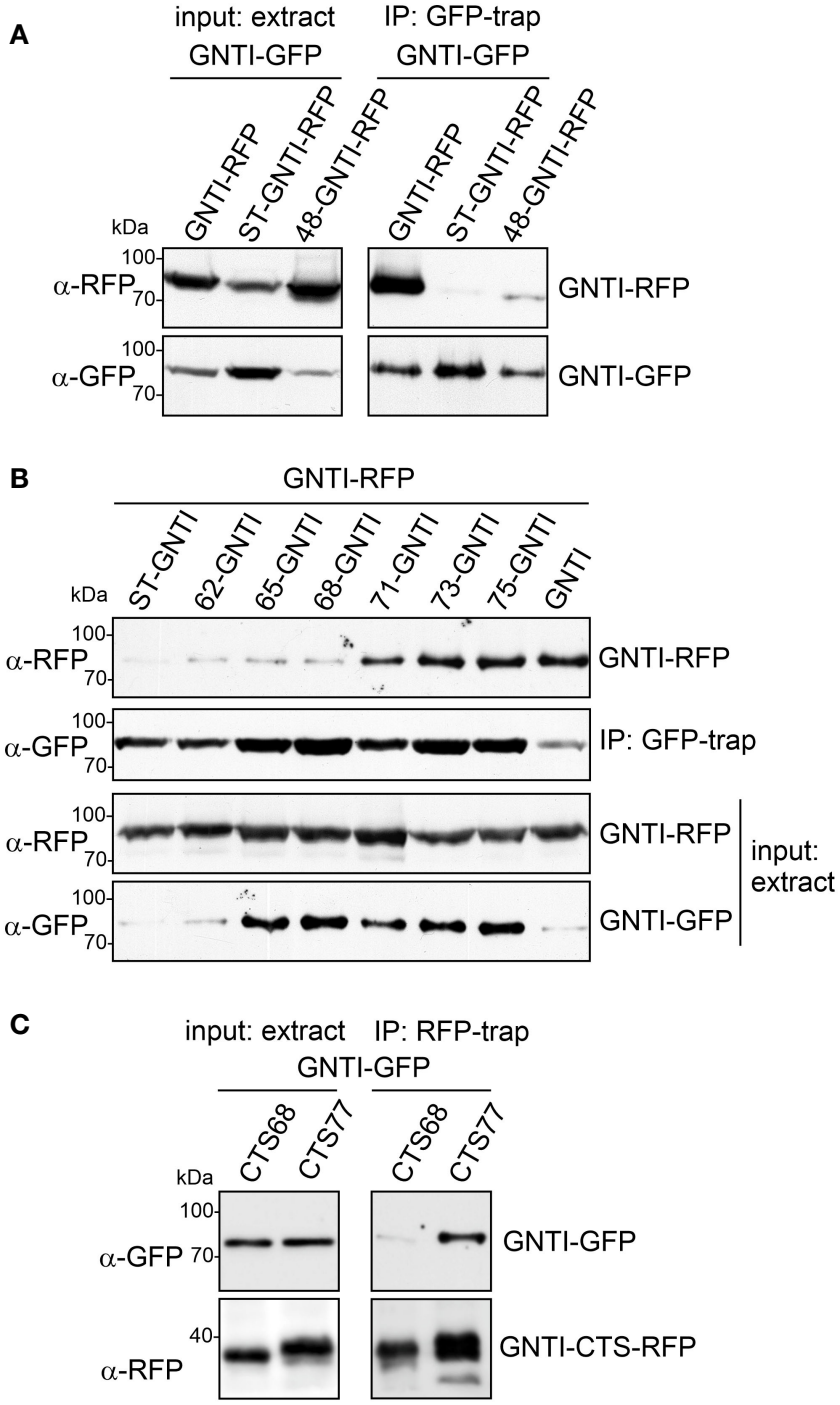
A region within the GNTI coiled-coil domain is required for homomeric complex formation. **(A)** Co-IP of GNTI-GFP with GNTI-RFP, ST-GNTI-RFP or 48-GNTI-RFP. **(B)** Co-IP of different GNTI-GFP stem deletion variants with GNTI-RFP. **(C)** Co-IP of GNTI-GFP co-expressed with GNTI-CTS77-RFP or GNTI-CTS68-RFP. All proteins were transiently expressed in *N. benthamiana*, and protein extracts were analyzed 2 days after infiltration.

To determine in more detail the amino acid motif involved in GNTI homomeric complex formation and its impact on complex N-glycan processing, we co-expressed stem-deleted GNTI variants (**Figure 2D**) with full-length GNTI and performed co-immunoprecipitations (co-IPs). A region within the stem is likely associated with the observed dominant-negative effect on N-glycan processing caused by binding to endogenous GNTI. While GNTI-RFP co-immunoprecipitated with GNTI-GFP, virtually no signal was detected for the control protein ST-GNTI-RFP, which carries the CTS region of rat α2,6-sialyltransferase (ST, Boevink et al., 1998), and a clearly weaker signal was found for the 48-GNTI-RFP variant, which has a 29 amino acid-long deletion of the stem region (**Figure 4A**).

GFP-trap purification of different GNTI variants with deletions in the stem region and subsequent immunoblotting with anti-RFP antibodies against full-length GNTI-RFP showed that this fusion and the truncated GFP variants of 75-GNTI, 73-GNTI, and 71-GNTI can still interact and form a homomeric complex (**Figure 4B**). The 68-GNTI variant with only the 68 amino acid-long CTS region binds comparatively weaker, showing that the removal of three additional amino acids (ALE in the sequence motif ALEEQMKRQ) abolishes to a large extent the protein-protein interaction. To confirm this, we carried out co-IP with only the CTS77 and CTS68 regions fused to RFP and investigated the interaction with full-length GNTI-GFP. Immunoblotting of RFP-trap purified proteins revealed the co-purification of GNTI-GFP when GNTI-CTS77-RFP was purified from total soluble protein. In contrast, GNTI-CTS68-RFP displayed only a faint interaction (**Figure 4C**).

### Overexpression of the GNTI-CTS77 region may interfere with the subcellular localization of native GNTI

**Figure 5:**
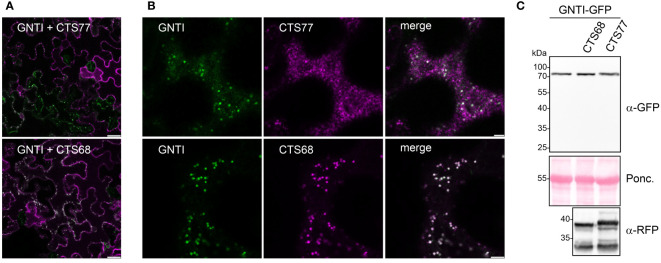
Co-expression of GNTI-GFP and GNTI-CTS77-RFP results in an aberrant subcellular localization. **(A, B)** Confocal microscopy analysis of GNTI-GFP (green) co-expressed with GNTI-CTS77-RFP (magenta) or GNTI-CTS68-RFP (magenta). Merged images show the overlay of the signals (in white). **(A)** A larger area of the leaf is shown. Scale bars = 25 µm. **(B)** A single-leaf epidermal cell is shown. Scale bars = 5 µm. **(C)** Immunoblot analysis of GNTI-GFP co-expressed with GNTI-CTS77-RFP or GNTI-CTS68-RFP harboring the GNTI-CTS region fused to RFP. Ponc., Ponceau S-stained membrane.

To gain further insight into the role of the overexpressed GNTI-CTS77 on endogenous GNTI, we analyzed the co-expressed GNTI variants by confocal microscopy. In contrast to GNTI-CTS68-RFP, GNTI-CTS77-RFP overexpression resulted in fewer cells with a fluorescent GFP signal, and aberrant fluorescence labeling was visible throughout the cells (**Figures 5A, B**). On immunoblots, GNTI-GFP levels were hardly affected in the presence of GNTI-CTS77-RFP and no degradation products could be detected (**Figure 5C**), suggesting that the main impact of the overexpression is on the subcellular localization of endogenous GNTI in the *cis*/medial Golgi.

**Figure 6:**
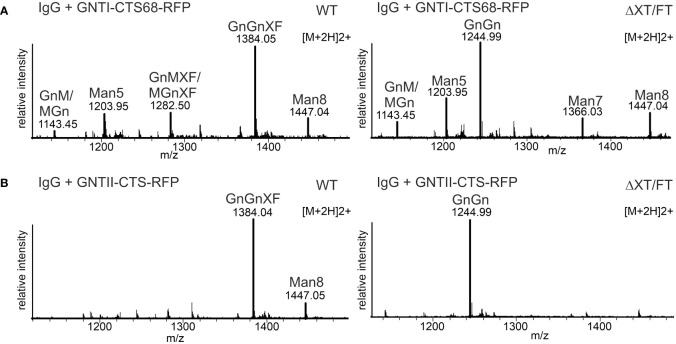
Expression of GNTI-CTS68-RFP results in low levels of Man_5_GlcNAc_2_. **(A)** MS spectra derived from the co-expression of a human IgG1 antibody with GNTI-CTS68-RFP in WT or in ΔXT/FT plants. **(B)** MS spectra of the peptide EEQYNSTYR derived from the co-expression of a human IgG1 antibody with the GNTII-CTS region fused to RFP (GNTII-CTS-RFP). The nomenclature for glycan abbreviations is in accordance with the ProGlycAn system (www.proglycan.com).

At the beginning of our study, we made the observation that glycoproteins such as IgG showed high levels of the glycan Man_5_GlcNAc_2_ when co-expressed with GNTI-CTS77-RFP. The same experiment carried out with GNTI-CTS68-RFP resulted in much lower levels of Man_5_GlcNAc_2_ N-glycans (**Figure 6A**). Consistent with the adverse effect of GNTI-CTS77 on the Golgi organization of *cis*/medial enzymes, an ER-retained GNTI-CTS77 region-containing fusion protein (C_AAA_TS) (Schoberer et al., 2009), chimeric CTS regions carrying the stem region of ST (GNTI-NNR-CTS) (Schoberer et al., 2014), and the CTS regions of GNTII or ST had negligible effects on the N-glycan processing and the formation of complex GnGn structures (**Figure 6B** and **Supplementary Figure S3**). Taken together, these data show that the stem region of the Golgi-located GNTI is responsible for the dominant-negative effect.

### GNTI-CTS77 expression results in the occurrence of predominantly mannosidic N-glycans on different recombinant glycoproteins

**Figure 7:**
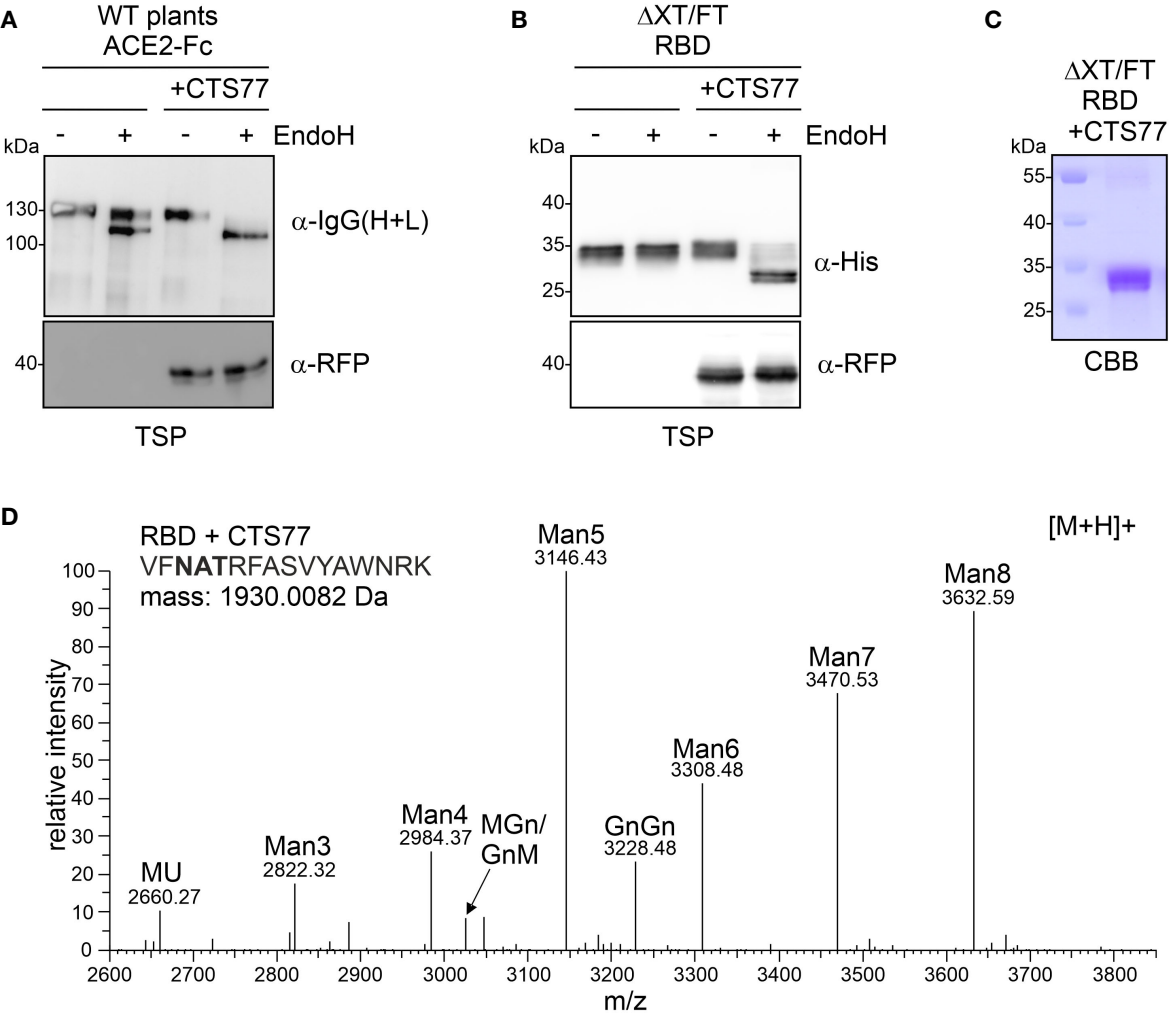
Transient co-expression of GNTI-CTS77-RFP in WT or ΔXT/FT plants generates mannosidic N-glycans on different recombinant glycoproteins. **(A)** Total soluble protein (TSP) extracts of ACE2-Fc expressed in WT *N. benthamiana* with (+CTS77) or without GNTI-CTS77-RFP and **(B)** TSP extract (obtained 3 days after the infiltration) of RBD expressed in ΔXT/FT were subjected to EndoH digestion, SDS-PAGE, and immunoblotting. **(C)** SDS-PAGE and Coomassie Brilliant Blue (CBB) staining of purified RBD co-expressed with GNTI-CTS77-RFP (+CTS77). **(D)** MS spectrum of the RBD glycopeptide carrying the N-glycosylation site N343 of the SARS-CoV-2 spike protein. RBD was co-expressed with GnTI-CTS77-RFP in ΔXT/FT plants and purified 4 days after infiltration. The nomenclature for glycan abbreviations is in accordance with the ProGlycAn system (www.proglycan.com).

Finally, we examined whether the dominant-negative effect of the GNTI-CTS77 region could be used as a novel glycoengineering tool to produce mannosidic N-glycans when transiently co-expressed with a recombinant glycoprotein. To this end, we expressed the RFP-tagged GNTI-CTS77 region together with different recombinant glycoproteins, such as human transferrin (hTF) (Castilho et al., 2011), human α1-antitrypsin (A1AT) (Castilho et al., 2014), human ACE2-Fc (Izadi et al., 2023), or the SARS-CoV-2 receptor binding domain (RBD) (Shin et al., 2021). Co-expression of GNTI-CTS77-RFP in wild-type *N. benthamiana* caused the accumulation of ACE2-Fc with exclusively EndoH-sensitive N-glycans (**Figure 7A**) and the formation of predominantly Man_5_GlcNAc_2_ structures on hTF and A1AT (**Supplementary Figure S4**). EndoH-sensitive mannosidic N-glycans were also detected on RBD when co-expressed with GNTI-CTS77-RFP in ΔXT/FT plants (**Figure 7B**). RBD expressed in ΔXT/FT carries typical GnGn-type complex N-glycans (Shin et al., 2021). To confirm the presence of mannosidic N-glycans on RBD co-expressed with GNTI-CTS77-RFP, we purified the protein from the apoplastic fluid and subjected the purified protein to MS-based glycopeptide analysis (**Figures 7C, D** and **Supplementary Figure S4**). Consistent with the strong effect on endogenous GNTI, we observed Man_5_GlcNAc_2_ as the predominant peak, followed by Man_8_GlcNAc_2_ and other mannosidic N-glycans. SPR analysis confirmed that RBD was functional in terms of binding to the ectodomain of the human ACE2 receptor (**Supplementary Figure S5**), suggesting no impact on RBD folding.

## Discussion

GNTI is a key enzyme of the N-glycan processing pathway as it initiates the formation of complex N-glycans (von Schaewen et al., 1993; Schachter, 2000), and GNTI deficiency leads to severe phenotypes in plants (Strasser, 2022). The tobacco GNTI protein is well characterized, and specific roles have been assigned to the cytoplasmic tail (Schoberer et al., 2009), the transmembrane domain (Schoberer et al., 2019), and the stem region (Schoberer et al., 2013). Here, we show that the CTS region contains a sequence motif (from amino acids 69 to 77) in the stem that causes interference with endogenous GNTI function when heterologously expressed. The organization of Golgi-resident glycosyltransferases such as GNTI is only partially understood in plants (Schoberer and Strasser, 2011; Schoberer et al., 2013; Schoberer et al., 2014; Schoberer et al., 2019). However, our previous studies have shown that GNTI can form both homo- and heteromeric protein complexes with MNS1 in the Golgi of *N. benthamiana* leaf epidermal cells (Schoberer et al., 2013; Schoberer et al., 2014). The occurrence of mannosidic N-glycans is consistent with a concomitant block of MNS1 activity by GNTI overexpression. Interestingly, overexpression of an MNS1-CTS region containing protein has a similar effect as GNTI-CTS overexpression. This suggests that the MNS1/GNTI complex is destabilized or mistargeted upon overexpression of a single interacting partner. How these complexes are organized within the Golgi is unknown, but our findings suggest that the stoichiometry and composition of the complex are controlled. Of note, MNS1 also has a predicted coiled-coil domain in the CTS region (**Supplementary Figure S2**), which is not found, for example, in Arabidopsis XYLT or GMII, which are enzymes that act later in the pathway and are not tightly linked to the MNS1/GNTI complex (Schoberer et al., 2013).

In a previous study, competition for localization in the same Golgi stack was observed between human and plant GNTI when both were co-expressed in Arabidopsis protoplasts (Henquet et al., 2010). Of note, the replacement of the CTS region of human GNTI with the CTS region (amino acids 1 to 102) of Arabidopsis GNTI resulted in a high degree of co-localization that was not observed when human GNTI was co-expressed with Arabidopsis GNTI. The observed incompatibility between human and Arabidopsis GNTI-CTS regions may also be related to specific sequence motifs in the GNTI stem region. The 77 amino acid-long tobacco GNTI-CTS region was also used in another study to target the catalytic domain of human β1,4-galactosyltransferase (GALT) to an earlier Golgi compartment (Vézina et al., 2009). Interestingly, a strong effect on complex N-glycan processing was observed when a recombinant antibody was co-expressed with the chimeric GNTI-GALT protein in *N. benthamiana*. It was suggested that the effect on N-glycan processing was related to GALT enzyme activity and that the interference with N-glycan processing was due to the relocation of the chimeric GNTI-GALT to an earlier Golgi compartment. However, the N-glycan shown then reflects more of a block of MNS1 activity, which could be a consequence of the disruption of the MNS1-GNTI Golgi complex as observed in our study.

Glycosylation of recombinant glycoproteins for therapeutic use or vaccination influences their function and efficacy and may also result in an unwanted immune response (Shi et al., 2022). To overcome potential issues with the presence of plant-specific complex N-glycan modifications like β1,2-xylose and core α1,3-fucose, numerous strategies have been employed in the past. Common approaches include gene knockout by homologous recombination (Koprivova et al., 2004), gene silencing (Strasser et al., 2008; Limkul et al., 2016), genome editing (Hanania et al., 2017; Mercx et al., 2017; Jansing et al., 2019; Jung et al., 2021; Göritzer et al., 2022; Kogelmann et al., 2023a), co-expression of enzymes that interfere with nucleotide sugar biosynthesis (Kogelmann et al., 2023b), or retention in the ER (Ko et al., 2003; Rosenberg et al., 2022). For whole plants such as *N. benthamiana*, most of these approaches typically involve plant transformation and are therefore time-consuming. The addition of KDEL-like peptides to the C-terminus for ER retention results in changes in the amino acid sequence of the recombinant protein, which could create an unwanted immunogenic epitope and may raise issues with approval authorities (Petruccelli et al., 2006). Compared to time-consuming plant transformation approaches, the transient overexpression of the tobacco GNTI-CTS region is simple, fast, and can be applied in wild-type plants to remove most of the plant complex N-glycans from recombinant glycoproteins, as shown for ACE2-Fc. The simplicity of the approach allows for easy generation of different glycoforms for structure-function studies and provides tools to produce recombinant glycoproteins with increased levels of mannosidic N-glycans, which are suitable as biopharmaceuticals for enzyme replacement therapy (He et al., 2012; Limkul et al., 2016; Jung et al., 2017). Importantly, expressed recombinant glycoproteins such as A1AT, hTF, and RBD with predominantly Man_5_GlcNAc_2_ N-glycans could be purified from intracellular fluid, showing that secretion is not affected. In terms of yield, most recombinant proteins were unaffected; only for RBD, the yield was consistently lower when GNTI-CTS77 was co-expressed. However, recombinant RBD carrying mannosidic N-glycans was functional in terms of receptor binding indicating that GNTI-CTS77 expression does not cause misfolding.

In summary, we have provided insights into the role of a conserved sequence motif in the stem region of plant GNTIs. Transient expression of GNTI-CTS carrying this motif can be used as a novel glycoengineering tool in plants to enable the simple production of mannosidic N-glycans on recombinant glycoproteins used for research or therapeutic applications.

## Materials and methods

### Cloning of constructs

The truncated *N. tabacum* GNTI-CTS regions were amplified by PCR (**Supplementary Table S1**), *Xba*I/*Bam*HI digested, and ligated into *Xba*I/*Bam*HI digested p46 to generate fusion proteins of the CTS region with the GNTI catalytic domain and GFP (Schoberer et al., 2014). The CTS48 *Xba*I/*Bam*HI digested fragment was also cloned into p49 (48-GNTI-RFP) (Schoberer et al., 2014). The p31-CTS68 (GNTI-CTS68-RFP) construct was generated by PCR amplification of the DNA sequence encoding the GNTI CTS region using the listed primer pair, *Xba*I/*Bam*HI digestion, and ligation into *Xba*I/*Bam*HI digested p31 (Schoberer et al., 2014). p31-GNTII-CTS (GNTII-CTS-RFP) was generated by amplification of the GNTII CTS region by PCR from *A. thaliana* cDNA, *Xba*I/*Bam*HI digestion, and ligation into *Xba*I/*Bam*HI digested p31. Expression vectors for fluorescent fusion proteins: p46-RRR (ST-GNTI-GFP) (Schoberer et al., 2014), p49-RRR (ST-GNTI-RFP) (Schoberer et al., 2014), p46-NNN (GNTI-GFP) (Schoberer et al., 2014), p49-NNN (GNTI-RFP) (Schoberer et al., 2014), p31-NNR (GNTI-NNR-CTS-RFP) (Schoberer et al., 2014), p31-NNN (GNTI-CTS77-RFP) (Schoberer et al., 2014), p20-MNS1-CTS (MNS1-CTS-GFP) (Liebminger et al., 2009), p20-XYLT-CTS (XYLT-CTS-GFP) (Schoberer et al., 2013), p31-MnII-CTS (GMII-CTS-RFP) (Schoberer et al., 2013), p31-GNTI-C_AAA_TS-RFP (GNTI-C_AAA_TS-RFP) (Schoberer et al., 2009) and pVKH18-En6:STmRFP (ST-CTS-RFP) (Renna et al., 2005; Schoberer et al., 2010) were described previously. Expression vectors for the glycoproteins human transferrin (hTF) (Castilho et al., 2011), human alpha-1-antitrypsin (A1AT) (Castilho et al., 2014), pEAQ-RBD-215 (RBD) (Shin et al., 2021), pEAQ-ACE2-Fc (ACE2-Fc) (Izadi et al., 2023), and 2G12 IgG1 antibody (Schähs et al., 2007) were available from previous studies.

### Transient expression and immunoblotting

All plant expression vectors were transformed into *Agrobacterium tumefaciens* (strain UIA143) (Farrand et al., 1989). Syringe-mediated agroinfiltration (OD_600 =_ 0.2) was used for transient expression in the leaves of 5-week-old *N. benthamiana* grown on soil under long-day conditions (16 h light/8 h dark) at 24°C. At the indicated time points, leaves were harvested from infiltrated plants, total soluble protein extracts were prepared, and they were subjected to SDS-PAGE followed by Coomassie Brilliant Blue staining or immunoblotting as previously described in detail (Shin et al., 2017). Fc-containing proteins were detected with anti-human IgG (H+L)-horseradish peroxidase antibody (Promega), GFP- and RFP-tagged proteins with anti-GFP (Roche) and anti-RFP (Proteintech) antibodies, respectively, and RBD with anti-His (Thermo Fisher Scientific) antibody. For deglycosylation, protein extracts were incubated with endoglycosidase H (EndoH, New England Biolabs) according to the manufacturer’s instructions. His-tagged RBD was purified from the collected intracellular fluid by loading onto a 5 ml HisTrap HP column (Cytiva), followed by elution with imidazole, dialysis, and concentration by ultracentrifugation, as described in detail previously (Göritzer et al., 2019).

### Co-immunoprecipitation

For the analysis of GNTI-GNTI protein interactions, GNTI-GFP was co-expressed with GNTI-RFP, 48-GNTI-RFP, and ST-GNTI-RFP, respectively, by infiltrating *N. benthamiana* leaves with agrobacterial suspensions carrying the different expression plasmids (OD_600 =_ 0.1 for all agrobacteria). Two days after infiltration, the GFP-tagged proteins were purified using GFP-Trap-A beads (Proteintech), and the co-purified protein was analyzed by immunoblotting with anti-RFP antibodies. GNTI-RFP was co-expressed with GNTI-GFP stem deletion variants and analyzed in the same manner. GNTI-GFP was co-expressed with p31-CTS68 (GNTI-CTS68-RFP) and p31-NNN (GNTI-CTS77-RFP), respectively, and RFP-tagged proteins were purified using RFP-Trap-A beads (Proteintech) and subjected to immunoblotting.

### LC-ESI-MS analysis

Plant-expressed IgG1 was purified from the protein extract by binding to rProtein A Sepharose™ Fast Flow (Cytiva) (Strasser et al., 2009). For A1AT and hTF, apoplastic fluid was isolated from infiltrated plants as previously described (Castilho et al., 2011). Purified IgG1 or apoplastic fluid was subjected to SDS-PAGE under reducing conditions, followed by CBB staining. Corresponding protein bands were excised from the gel, destained, carbamidomethylated, in-gel trypsin digested, and analyzed by liquid chromatography-electrospray ionization mass spectrometry (LC-ESI-MS), as previously described in detail (Stadlmann et al., 2008). Purified RBD was S-alkylated with iodoacetamide and digested in solution with the endoproteinases LysC (Roche) and GluC (Promega) (Shin et al., 2021). The digested samples were analyzed using an Orbitrap Exploris 480 (Thermo Fisher Scientific) (Izadi et al., 2023) and the data obtained were analyzed using the Skyline software, version 22.2 (Maclean et al., 2010).

### Confocal imaging of fluorescent protein fusions

Leaves of 5-week-old *N. benthamiana* were infiltrated with agrobacterium suspensions carrying binary plant expression vectors for expression of GFP- or RFP-tagged proteins (OD_600 =_ 0.1 for GNTI-GFP, 68-GNTI-GFP, 48-GNTI-GFP, and p48-MUR3). Confocal images were acquired 2 days post-infiltration on a Leica SP5 II confocal microscope (Schoberer et al., 2019) using the Leica LAS AF software system (http://www.leica.com). Dual-color image acquisition of cells expressing both GFP and RFP was performed simultaneously. Post-acquisition image processing was performed in Adobe Photoshop CS5.

### SPR

Surface plasmon resonance (SPR) experiments were performed using a Biacore T200 (Cytiva). All assays were performed in HBS-EP running buffer (Cytiva) at 25°C. To determine the binding kinetics between the RBD-215 variants (MW = ∼24 kDa without glycans) and ACE2-Fc (MW = 189,8 kDa, produced in HEK293 cells) (Castilho et al., 2021), a CM5 chip was coated with 150 pg of Protein A via amine coupling in the active cell (5500 RU). The reference flow cell was left blank without Protein A. The ligand ACE2-Fc was immobilized non-covalently at 2.63 nM. The analytes, RBD-215 variants, were injected across the two flow cells at a range of five concentrations (200, 80, 32, 12.8, and 5.12 nM) prepared by serial 2.5-fold dilutions at a flow rate of 30 μL/min using a single-cycle kinetics program. Prior to SPR measurements, the protein concentration for each sample was verified by averaging five independent measurements taken with a NanoDrop spectrophotometer in UV mode (at 280 nm using sample-specific extinction coefficients). Running buffers were also injected using the same program for background subtraction. The chip was regenerated with 10 mM glycine-HCl, pH 1.7, after each measurement. The ACE2-Fc ligand was captured at every run. The data were fitted to a 1:1 binding model using Biacore T200 Evaluation Software, version 3.1. The sensorgram was plotted using GraphPad Prism, version 9.0.

## Data availability statement

The original contributions presented in the study are included in the article/**Supplementary Material**, further inquiries can be directed to the corresponding author/s.

## Author contributions

JS: Conceptualization, Formal Analysis, Investigation, Writing – original draft. SI: Investigation, Formal Analysis, Writing – review & editing. CK: Investigation, Formal Analysis, Writing – review & editing. UV: Investigation, Formal Analysis, Writing – review & editing. JK-B: Investigation, Formal Analysis, Writing – review & editing. VR: Formal Analysis, Investigation, Writing – review & editing. CG-G: Investigation, Methodology, Formal Analysis, Writing – review & editing. AC: Conceptualization, Formal Analysis, Funding acquisition, Resources, Writing – original draft. RS: Conceptualization, Data curation, Formal Analysis, Funding acquisition, Supervision, Writing – original draft.
