# Supplementary material for: The tobacco GNTI stem region harbors a strong motif for homomeric protein complex formation
Source: Front Plant Sci. 2023 Nov 28;14:1320051. doi: 10.3389/fpls.2023.1320051 (PMC10715278; doi:10.3389/fpls.2023.1320051)
Supplement: Supplementary file 1 [file DataSheet_1.pdf]

Supplementary Material

The tobacco GNTI stem region harbors a strong motif for homomeric protein complex formation

Jennifer Schoberer[1], Shiva Izadi[1], Carolina Kierein[1], Ulrike Vavra[1], Julia König-Beihammer[1], Valentina Ruocco[1], Clemens Grünwald-Gruber[2], Alexandra Castilho[1], Richard Strasser[1]*

[1]Department of Applied Genetics and Cell Biology, University of Natural Resources and Life Sciences, Vienna, Austria

[2]Core Facility Mass Spectrometry, University of Natural Resources and Life Sciences, Vienna, Austria

*Correspondence:

Richard Strasser

Email: richard.strasser@boku.ac.at

Keywords: cell biology, glycoengineering, glycosylation, Golgi apparatus, protein-protein interaction, recombinant protein

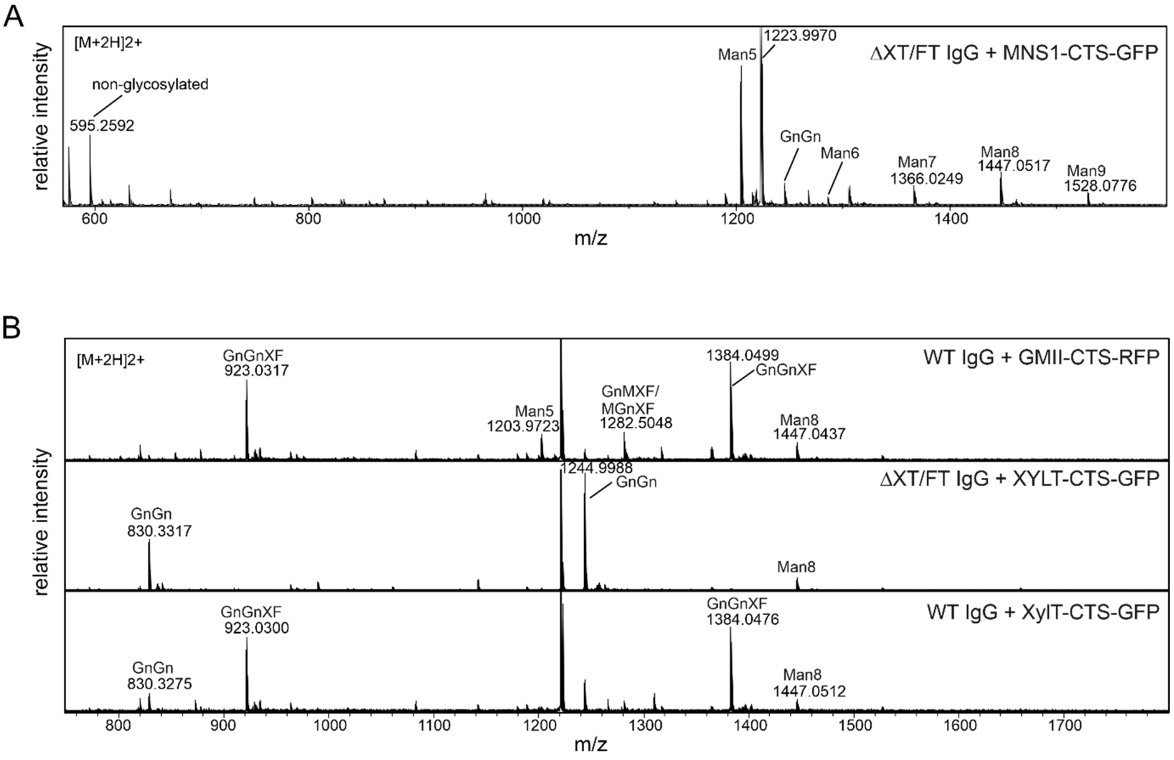

Supplemental Figure S1. MS-spectra of glycopeptides (peptide sequence: EEQYNSTYR derived by trypsin digestion of the heavy chain polypeptide from human IgG1). (**A**) IgG co-expressed with MNS1-CTS-GFP (Liebminger et al., 2009) or (**B**) IgG co-expressed with GMII-CTS-RFP or XYLT-CTS-GFP (Schoberer et al., 2013). Nomenclature for glycan abbreviations is in accordance with the ProGlycAn system (www.proglycan.com). Please note, the major peak at ca. 1224 represents the lock mass and is not related to any glycan structure.

A

NtGnTI

1	**MRGYKF**CCDF	RYLLILAAVA	FIYIQMRLFA	TQSEYADRLA	AAIEAENHCT	50
51	SQTRLLIDQI	SQQQGRIVA**L**	**EEQ**MKRQDQE	CRQLRALVQD	LESKG**IKKL**I	100
101	GNVQMPVAAV	VVMACNRADY	LEKTIKSILK	YQISVAPKYP	LFISQDGSHP	150
151	DVRKLALSYD	QLTYMQHLD**F**	**EPVHTERPGE**	LIAYYKIARH	YKWALDQLFY	200
201	KHNFSRVIIL	EDDMEIAPDF	FDFFEAGATL	LDRDKSIMAI	SSWNDNGQMQ	250
251	FVQDPYALYR	SDFFPGLGWM	LSKSTWDELS	PKWPKAYWDD	WLRLKENHRG	300
301	RQFIRPEVCR	SYNFGEHGSS	LGQFFKQYLE	PIKLNDVQVD	WKSMDLSYLL	350
351	EDNYVKHFGD	LVKKAKPIHG	ADAVLKAFNI	DGDVRIQYRD	QLDFEDIARQ	400
401	FGIFEEWKDG	VPRAAYKGIV	VFRYQTSRRV	FLVGPDS**LQQ**	**LGNEDT**	450

B

MNS1

1	**MARSRSISGY**	**GIW**KYLNPAY	YLRRPRRLAL	LFIVFVSVSM	LVWDRINLAR	50
51	**EHEVEVFKLN**	**EEVSRLEQML**	**EELNGGVGNK**	**PLKTLKDAPE**	**D**PVDKQRRQK	100
101	VKEAMIHAWS	SYEKYAWGKD	ELQPRTKDGT	DSFGGLGATM	VDSLDTLYIM	150
151	GLDEQFQKAR	EWVASSLDFD	KDYDASMFET	TIRVVGGLLS	AYDLSGDKMF	200
201	LEKAKDIADR	LLPAWNTPTG	IPYNIINLRN	**GNAHNPSWAA**	**GGDSILA**DSG	250
251	TEQLEFIALS	QRTGDPKYQQ	KVEKVITELN	KNFPADGLLP	IYINPDNANP	300
301	SYSTTTFGAM	GDSFYEYLLK	VWVQGNKTSA	VKPYRDMWEK	SMKGLLSLVK	350
351	KSTPSSFTYI	CEKNGNNLID	KMDELACFAP	GMLALGASGY	GPDEEKKFLS	400
401	LAGELAWTCY	NFYQSTPTKL	AGENYFFTAG	QDMSVGTSWN	ILRPETVESL	450
451	FYLWRLTGNK	TYQEWGWNIF	QAFEKNSRVE	SGYVGLKDVN	TGAKDNKMQS	500
501	FFLAETLKYL	YLLFSPSSVI	SLDEWVFNTE	AHPLKIVARN	DPRKPTI**ALR**	550
551	**QRKFGHQINV**					600

C

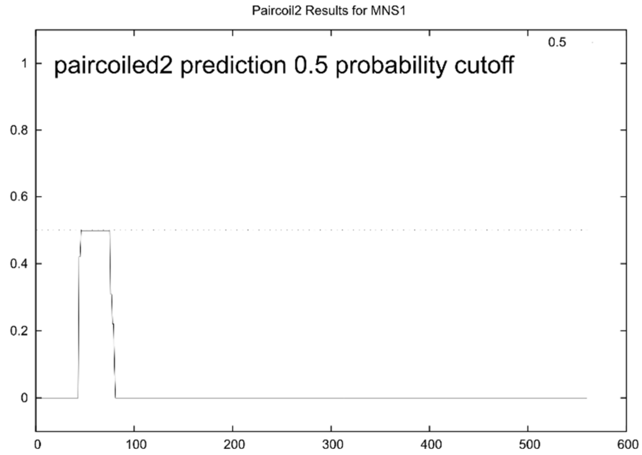

Supplemental Figure S2. Prediction of disordered regions in (**A**) *N. tabacum* GNTI and (**B**) *A. thaliana* MNS1 using PrDOS - Protein DisOrder prediction system (https://prdos.hgc.jp/cgi-bin/top.cg). Potentially disordered regions are shown in red. (**C**) Prediction of coiled-coil domains in MNS1 using Paircoil2 (https://cb.csail.mit.edu/cb/paircoil2/paircoil2.html).

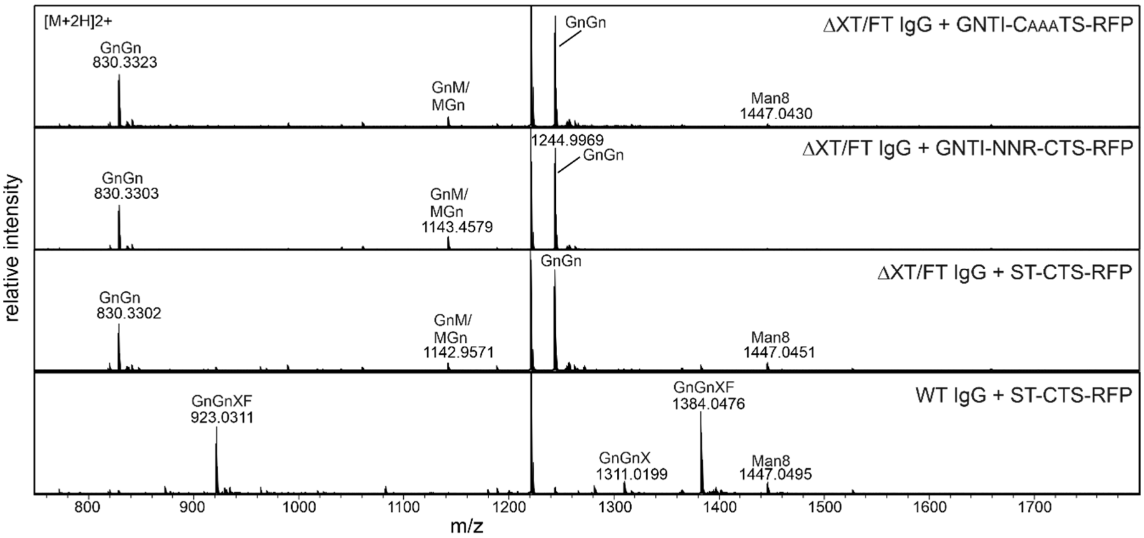

Supplemental Figure S3. MS-spectra of glycopeptides (peptide sequence: EEQYNSTYR derived by trypsin digestion of the heavy chain polypeptide from human IgG1). IgG co-expressed with GNTI-CAAATS-RFP (ER-retained GNTI-CTS77-region due to mutations in the ER export signal within the cytoplasmic tail, the transmembrane and stem region is the same like in GNTI-CTS77) (Schoberer et al., 2009); IgG co-expressed with GNTI-NNR-CTS-RFP (GNTI stem region exchanged with stem from ST, CT region from GNTI) (Schoberer et al., 2014); IgG co-expressed with ST-CTS-RFP (ST-CTS region) (Schoberer et al., 2010). Nomenclature for glycan abbreviations is in accordance with the ProGlycAn system (www.proglycan.com). Please note, the major peak at ca. 1224 represents the lock mass and is not related to any glycan structure.

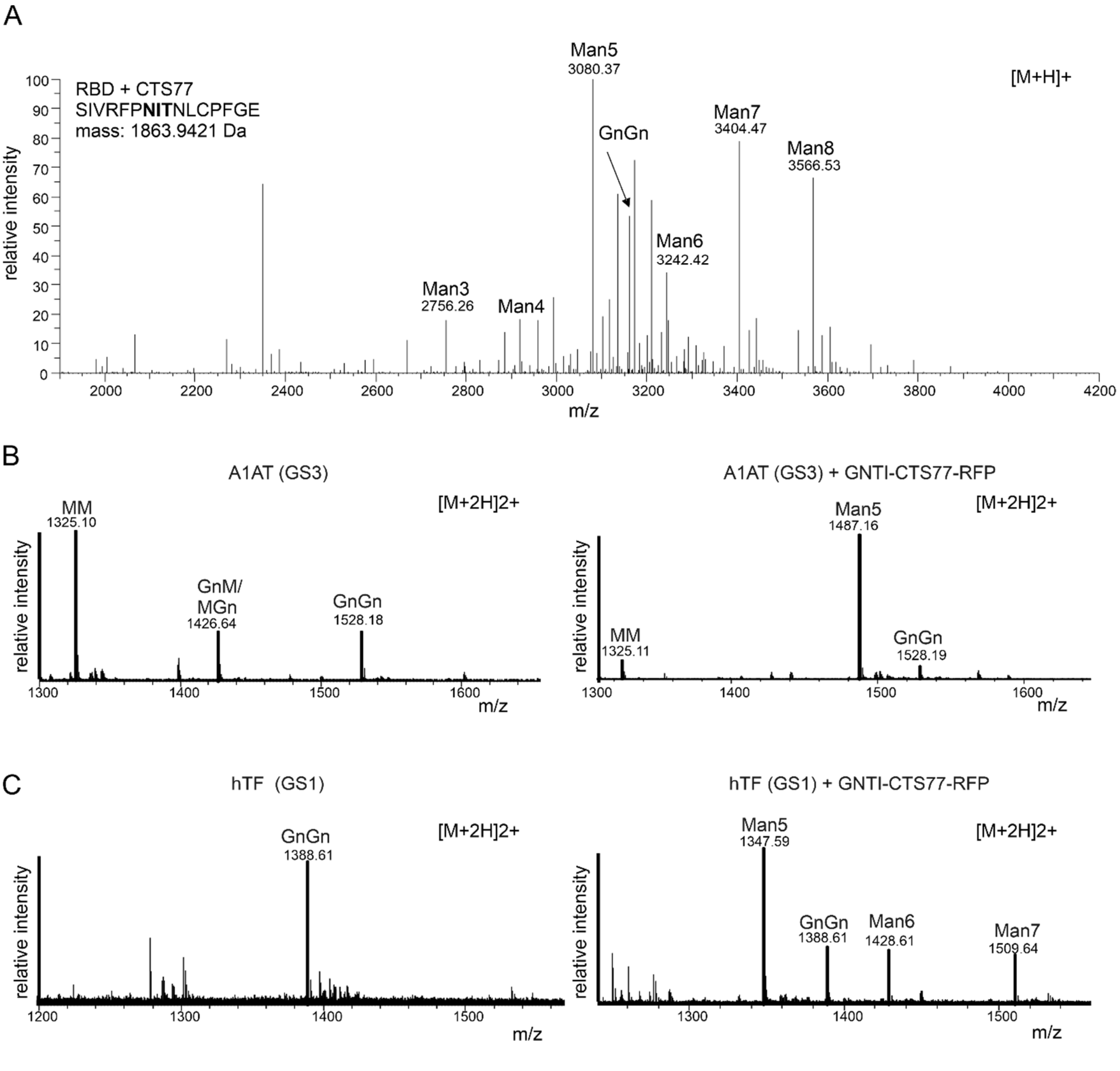

Supplemental Figure S4. MS-spectra of (**A**) the glycopeptide carrying N-glycosylation site N331 from the SARS-CoV-2 RBD (SIVRFPNITNLCPFGE, mass 1863.94 $[M+H]^+$) co-expressed in *N. benthamiana* ΔXT/FT with GNTI-CTS77-RFP or (**B**) human α1-antitrypsin (A1AT) glycopeptide carrying glycosylation site 3 (GS3: YLGNATAIFFLPDEGK, mass 1755.89 $[M+H]^+$) or (**C**) human transferrin (hTF) glycopeptide carrying glycosylation site 1 (GS1: GLVPVLAENYNK, mass 1476.75 $[M+H]^+$) both co-expressed with GNTI-CTS77-RFP. Nomenclature for glycan abbreviations is in accordance with the ProGlycAn system (www.proglycan.com).

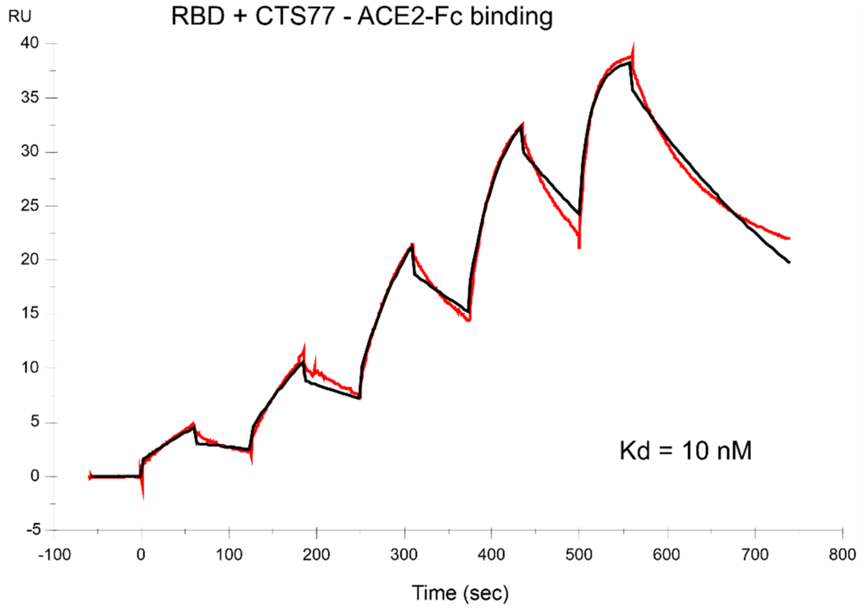

Supplemental Figure S5. Sensorgram from SPR spectroscopy in multi-cycle kinetic experiments for ACE2-Fc and RBD which was co-expressed with GNTI-CTS77-RFP and purified from the apoplastic fluid.

Supplemental Table S1. List of primers used for CTS region amplification.

CTS region	Forward primer	Reverse primer
GNTI-CTS48	5′-TATA**TCTAGA**ATGAGAGGGTACAAGTTTTGCTGTG-3′	5′-TATA**GGATCC**GTGATTTTCTGCTTCAATTGCAG-3′
GNTI-CTS62	5′-TATA**TCTAGA**ATGAGAGGGTACAAGTTTTGCTGTG-3′	5′-TATA**GGATCC**CTGGCTAATCTGGTCAATAAGCAA-3′
GNTI-CTS65	5′-TATA**TCTAGA**ATGAGAGGGTACAAGTTTTGCTGTG-3′	5′-TATA**GGATCC**TCCTTGCTGCTGGCTAATCTGGT-3′
GNTI-CTS68	5′-TATA**TCTAGA**ATGAGAGGGTACAAGTTTTGCTGTG-3′	5′-TATA**GGATCC**AACTATTCTTCCTTGCTGCTGGC-3′
GNTI-CTS71	5′-TATA**TCTAGA**ATGAGAGGGTACAAGTTTTGCTGTG-3′	5′-TATA**GGATCC**TTCAAGAGCAACTATTCTTCCTT-3′
GNTI-CTS73	5′-TATA**TCTAGA**ATGAGAGGGTACAAGTTTTGCTGTG-3′	5′-TATA**GGATCC**TTGTTCTTCAAGAGCAACTATTCT-3′
GNTI-CTS75	5′-TATA**TCTAGA**ATGAGAGGGTACAAGTTTTGCTGTG-3′	5′-TATA**GGATCC**CTTCATTTGTTCTTCAAGAGCAAC-3′
GNTII-CTS	5′-ATA**TCTAGA**ATGGCAAATCTTTGGAAGAAGC-3′	5′-TATA**GGATCC**CAAAGCAGTTCTAGGTACAGA-3′

The *Xba*I/*Bam*HI restriction sites are shown in bold.
